# Quantification of *Plasmodium knowlesi* versus *Plasmodium falciparum* in the rhesus liver: implications for malaria vaccine studies in rhesus models

**DOI:** 10.1186/s12936-020-03385-4

**Published:** 2020-08-31

**Authors:** Melanie J. Shears, Annette M. Seilie, B. Kim Lee Sim, Stephen L. Hoffman, Sean C. Murphy

**Affiliations:** 1 Department of Laboratory Medicine and Pathology, University of Washington, 750 Republican St., F870, Seattle, WA 98109 USA; 2 Center for Emerging and Re-emerging Infectious Diseases, University of Washington, 750 Republican St., Seattle, WA 98109 USA; 3 Sanaria, Inc., 9800 Medical Center Drive, Suite A209, Rockville, MD 20850 USA; 4 Washington National Primate Research Center, University of Washington, 1959 NE Pacific St., Seattle, WA 98195 USA

**Keywords:** Rhesus macaque, *P. falciparum*, *P. knowlesi*, Sporozoite, Vaccine, PfSPZ, PkSPZ

## Abstract

**Background:**

Rhesus macaques are valuable pre-clinical models for malaria vaccine development. The *Plasmodium knowlesi*/rhesus and *Plasmodium falciparum*/rhesus models are two established platforms for malaria vaccine testing, and both have previously been used to assess live-attenuated sporozoite vaccines. However, there is evidence that the susceptibility of the rhesus liver to *P. knowlesi* versus *P. falciparum* sporozoites likely differs, potentially complicating comparisons between these two platforms.

**Methods:**

To quantify the differing susceptibility of rhesus to *P. knowlesi* and *P. falciparum* sporozoites, animals were infected by direct venous inoculation of purified, cryopreserved wild-type *P. knowlesi* sporozoites (PkSPZ) or *P. falciparum* sporozoites (PfSPZ). The entire liver was collected 5 days post-infection, and parasite burden in each liver lobe was quantified using an ultrasensitive *Plasmodium* 18S rRNA RT-PCR biomarker assay. The potential of using 18S rRNA copy number in the rhesus liver to directly measure the efficacy of vaccines targeting *P. falciparum* sporozoites and liver stages was also theoretically evaluated.

**Results:**

Infection of rhesus with a high dose of PkSPZ led to consistently high burden liver stage infections (range 9.5–10.1 log_10_ copies 18S rRNA/g of liver), with similar amounts of parasite 18S rRNA detected in every liver lobe. Inoculation of rhesus with high doses of PfSPZ led to more variable, lower liver burdens (range 4.9–6.6 log_10_ copies 18S rRNA/g of liver in infected lobes), with parasite 18S rRNA below the limit of detection in some liver lobes. The low signal and heterogeneity of liver burden in the PfSPZ-infected animals indicates that even this extremely sensitive molecular assay cannot be used to assess reliably vaccine efficacy in the *P. falciparum*/rhesus platform.

**Conclusions:**

Detection of 18S rRNA in the liver following high dose intravenous PfSPZ confirmed that rhesus are modestly susceptible to wild-type *P. falciparum* sporozoites. However, comparison of 18S rRNA RT-PCR biomarker signal indicates that the *P. falciparum* liver burden was 3–5 logs lower than in PkSPZ-infected animals. Quantification of this difference in liver stage burden will help guide and interpret data from pre-clinical studies of live-attenuated sporozoite vaccines in rhesus models.

## Background

Malaria control efforts would benefit enormously from development of a highly effective and easily deployable malaria vaccine that achieves durable sterile protection against *Plasmodium falciparum* malaria. The *Plasmodium* sporozoite and liver stages are considered strategic targets for intervention, and a number of strategies designed to block these stages have been developed including protein subunit vaccines, viral-vectored vaccines, and live-attenuated vaccines such as radiation-attenuated sporozoites and genetically- or chemically-attenuated parasite vaccines [1].

Despite numerous protective vaccine candidates being characterized in rodent malaria models, translating such findings to humans has been difficult and many clinical trials have shown underwhelming efficacy [2–4], suggesting that rodent models may not faithfully predict vaccine efficacy in humans. To date, purified, cryopreserved, live-attenuated *P. falciparum* sporozoite (PfSPZ) vaccines have provided the best vaccine efficacy in humans, achieving up to 100% sterile protection against controlled human malaria infections in clinical trials [5–7]. However, although there has been significant progress in this area [8], to achieve the goals of durable, high level vaccine efficacy at low cost, there is still a need to develop new malaria vaccine strategies and translationally-relevant models for their evaluation.

Rhesus macaques are valuable pre-clinical models that allow for assessment of malaria vaccine safety, immunogenicity and protective efficacy. Rhesus are considered excellent models of the human immune system [9], and the availability of immunological tools, together with the ability to study the liver and other tissues inaccessible in humans, makes them ideally suited for translationally-relevant vaccine research [10]. Both *Plasmodium knowlesi* and *P. falciparum* have been used in rhesus for malaria vaccine testing. *Plasmodium knowlesi* parasites naturally infect non-human primates and cause zoonotic malaria infections in humans [11]. The *P. knowlesi*/rhesus model can be used to evaluate malaria vaccine immunogenicity and efficacy, and because *P. knowlesi* is highly virulent in rhesus, provides a stringent model for assessing vaccine strategies prior to human trials [11, 12]. The *P. falciparum*/rhesus model is an alternative platform that allows direct evaluation of vaccines targeting this parasite species. However, since rhesus do not support *P. falciparum* blood stage infections [13], this platform is typically used to assess vaccine immunogenicity as animals are not amenable to challenge with a blood stage efficacy endpoint [14, 15].

For live-attenuated sporozoite vaccines against *P. falciparum*, which almost certainly rely on successful sporozoite invasion in the liver for antigen delivery [15], the use of rhesus for vaccine assessment critically hinges on their susceptibility to sporozoite infection. Fortunately, despite being unable to support the *P. falciparum* blood stage, previous data have demonstrated that rhesus have some susceptibility to *P. falciparum* sporozoite infection in the liver. Data from two independent studies have shown that *P. falciparum* sporozoites can invade and develop in rhesus primary hepatocytes in vitro, albeit with less efficiency than *P. falciparum* in human hepatocytes or *P. knowlesi* in rhesus hepatocytes [15, 16]. These findings validated the use of rhesus for testing the immunogenicity of live-attenuated PfSPZ vaccines, and enabled pivotal non-human primate studies [15] that guided subsequent clinical trials [5, 17]. Complementary studies in the *P. knowlesi*/rhesus challenge model contributed further insight into the immunogenicity of live-attenuated sporozoite vaccines [18] and allowed for more definitive identification of correlates of protection in the liver [19]. Rhesus have thus been key for elucidating mechanisms of vaccine-induced immunity that are expected to translate to humans. However, integrating data from these two platforms would undoubtedly be aided by a greater understanding of rhesus susceptibility to *P. falciparum* versus *P. knowlesi* sporozoites in vivo.

Recent data are beginning to shed light on this question. In a prior study, rhesus were inoculated intravenously with a high dose (6.5 × 10^6^) of freshly-dissected wild-type *P. falciparum* sporozoites and parasite liver burden was quantified 3 or 6 days later using ultra-sensitive 18S rRNA RT-PCR [20]. Parasite 18S rRNA was detected in the rhesus liver at both time points, with RNA copy number increasing 100-fold between days 3 and 6 [20]. This demonstrated measurable *P. falciparum* liver burden could be supported in rhesus, and provided the first quantification of rhesus susceptibility to *P. falciparum* sporozoites in vivo. These data also suggested the possibility of quantifying *P. falciparum* 18S rRNA in the rhesus liver to directly measure efficacy of vaccines targeting sporozoites and liver stages. Here, this study aims to extend upon this previous work by directly comparing the susceptibility of rhesus to wild-type *P. falciparum* versus *P. knowlesi* sporozoites in vivo, and theoretically evaluating the potential to use measurements of *P. falciparum* 18S rRNA in the liver as a vaccine efficacy endpoint.

## Methods

### Study design and rationale

Juvenile male and female Indian origin rhesus macaques (*Macaca mulatta*) were group-housed at the Washington National Primate Research Center (WaNPRC). Animals were malaria-naïve and tested at baseline for C-reactive protein (CRP) and complete blood count (CBC) to evaluate their level of systemic immune activation and overall health. Individuals were randomized into three treatment groups by sex and weight. All animals were infected by direct venous inoculation (DVI) of cryopreserved sporozoites at day 0. The dose and species of sporozoite used differed by treatment group as follows: Group A—1 × 10^6^ aseptic, purified, cryopreserved wild-type *P. falciparum* sporozoites (PfSPZ; two animals), Group B—5 × 10^6^ PfSPZ (two animals), and Group C—0.5 × 10^6^ purified cryopreserved, wild-type *P. knowlesi* sporozoites (PkSPZ; two animals). Sporozoite doses were chosen to reflect high doses based on published studies [20], with the final number determined by sporozoite availability. Animals were humanely euthanized at day 5 post challenge, at precisely 120 h post-challenge to the hour. This time was selected as it was expected to reliably precede parasite egress from the liver [21, 22]. The number of animals used was driven by budget constraints; the study was not designed for statistical power and, because of this, statistical tests are not reported between groups.

### Cryopreserved sporozoites

PfSPZ and PkSPZ were produced by Sanaria as described [5, 6]. Both were purified and cryopreserved, and the PfSPZ were aseptic. Sanaria’s aseptic, purified, cryopreserved PfSPZ have been shown to cause asexual erythrocytic stage infection in 100% of malaria-naïve human subjects at a dose of 3.2 × 10^3^ by DVI [6]. Purified, cryopreserved PkSPZ have been shown to cause asexual erythrocytic stage infection in 100% of malaria-naïve rhesus macaques at a dose of 0.5 × 10^3^ by DVI (S. Chakravarty, unpublished). PfSPZ were vialed at a 1 × 10^6^ PfSPZ per vial, and PkSPZ were vialed at 5 × 10^4^ PkSPZ per vial. Vials from a single PfSPZ batch and single PkSPZ batch were used. Separate vials of PfSPZ or vials of PkSPZ were used for each animal. PfSPZ and PkSPZ products were shipped, stored, thawed, diluted, and administered in strict accordance with Sanaria protocols. Sporozoites were diluted into 1% human serum albumin (CSL-Behring, #44206-251-10) in sterile USP grade phosphate buffered saline (RMBio, #BSS-PBS-1X6). Direct venous inoculation of diluted sporozoites was performed using a syringe into the saphenous vein under ketamine and dexmedetomidine sedation. Correct entry of the syringe into the vein was confirmed by pulling back on the syringe plunger to observe a return of blood prior to administration of the sporozoites.

### Blood and liver tissue processing

Blood for *Plasmodium* 18S rRNA RT-PCR was drawn prior to euthanasia into ETDA tubes. Blood was stored briefly at room temperature then 1 mL was added to 19 mL of NucliSENS lysis buffer (bioMérieux, #28013) and mixed to achieve homogeneous lysis. Liver tissue was collected after euthanasia into RPMI media (Gibco, #22400) supplemented with Penicillin–Streptomycin–Glutamine (Gibco, #10378-016) and 10% heat-inactivated fetal bovine serum (Sigma, #4135). The gall bladder was removed by dissection, and the livers were divided into four lobes as follows: left lateral lobe (LLL), left medial lobe (LML), right medial lobe plus caudate lobe (RML + C), and right lateral lobe (RLL). Lobes were individually weighed, then separately homogenized in 100 mL of NucliSENS lysis buffer with an immersion blender. Multiple immersion blenders were used with bleach and ethanol cleanings to eliminate carryover between animals. Lysed blood and liver homogenates were stored at − 80 °C until extraction.

### Nucleic acid extraction and *Plasmodium* 18S rRNA RT-PCR

Lysed blood and total liver lobe homogenates were thawed and further diluted for a final concentration of 50 µL whole blood or 50 mg liver to 2 mL of lysis buffer, of which 1 mL of lysate was processed in duplicate by the Abbott m2000sp. using the mSample RNA preparation kit (Abbott Molecular). Extracted RNA were subject to *Plasmodium* 18S rRNA quantitative reverse transcription-PCR (qRT-PCR) using the SensiFAST™ Probe Lo-Rox One-Step Kit (Bioline #BIO-78005) as previously described [23] but with locked nucleic acid analogs [+_] on the Pan-*Plasmodium* primers (Forward PanDDT1043F19: AAAGTTA[+A]GGGA[+G][+T]GAAGA, Reverse PanDDT1197R22: AA[+G]ACTTTGATTTCTC[+A]TAAGG; Qiagen) in addition to a quencher modification on the *P. falciparum* probe (5′-[6-FAM]-ATTTATTCAGTAATCAAATTAGGAT-3′ [Black Hole Quencher 1 PLUS]; LGC BioSearch Technologies) to permit an increased annealing temperature of 54 °C for improved assay specificity. Each specimen was quality controlled with a TATA-Binding Protein mRNA RT-PCR, and each qRT-PCR run was monitored with well-characterized run controls consisting of human whole blood samples. Results were quantified using an Armored RNA calibrator standard that encodes the *P. falciparum* 18S rRNA (Asuragen).

## Results

**Fig. 1:**
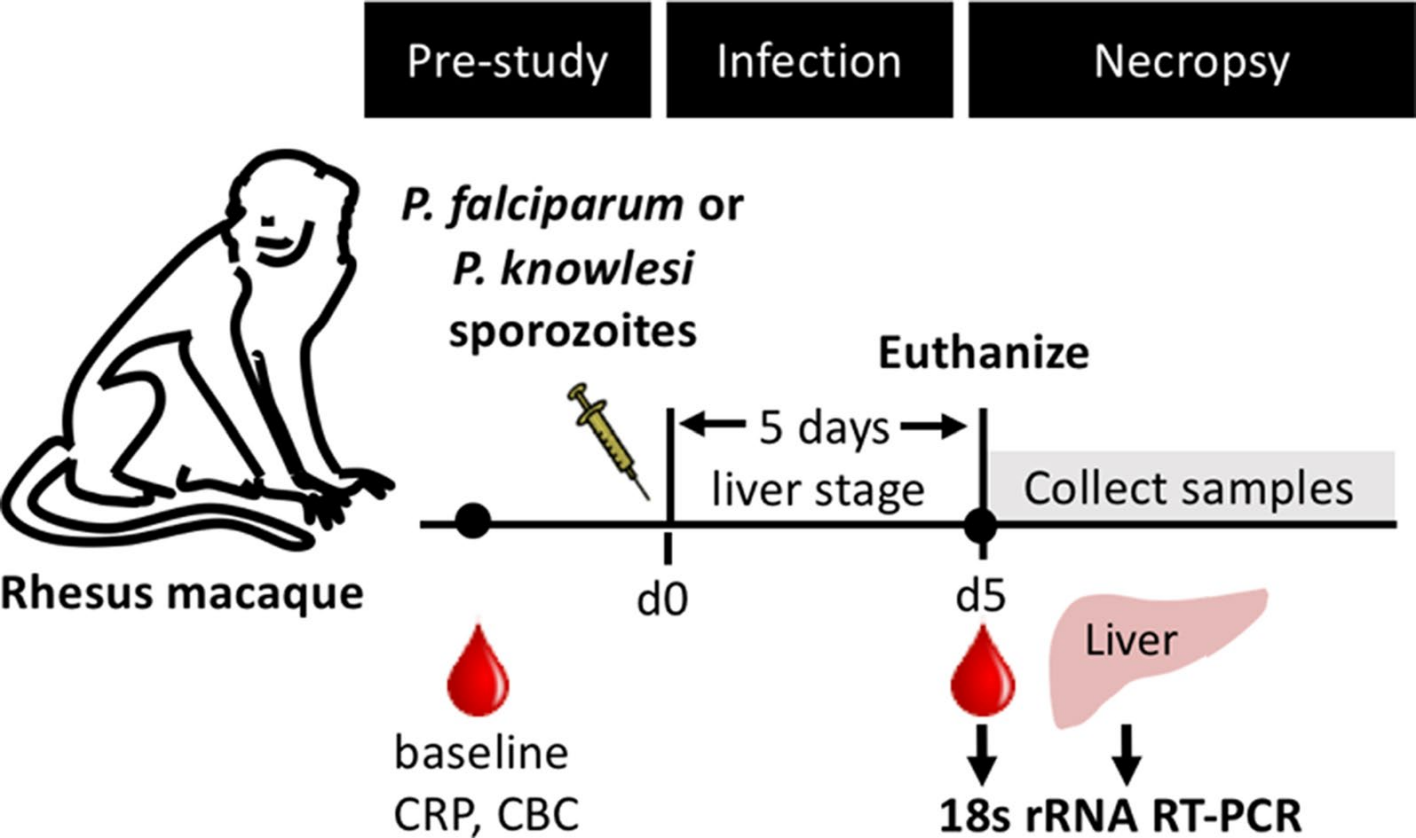
Study design. Rhesus macaques underwent baseline blood tests for C-reactive protein (CRP) and complete blood count (CBC). At day 0, animals were administered a high dose of PfSPZ or PkSPZ by direct venous inoculation. At day 5, blood was drawn, and animals were humanely euthanized to collect the entire liver. Blood and liver samples were then processed to quantify parasite burden using the ultra-sensitive 18S rRNA RT-PCR biomarker assay

The study was designed to directly compare the susceptibility of the rhesus liver to wild-type *P. falciparum* and *P. knowlesi* sporozoites in vivo (Fig. 1). Blood was drawn from animals at baseline for CRP and CBC to evaluate their health, and animals were inoculated at day 0 by DVI of either *P. falciparum* and *P. knowlesi* sporozoites according to their treatment group. Two animals per group were inoculated with sporozoites as follows: Group A—1 × 10^6^ PfSPZ, Group B—5 × 10^6^ PfSPZ, Group C—0.5 × 10^6^ PkSPZ. Groups A and B were designed to assess the dose-dependence of *P. falciparum* 18S rRNA liver burden following sporozoite inoculation, and to identify if either dose would theoretically allow for measurement of parasite 18S rRNA in the liver as a potential vaccine efficacy endpoint. Group C was designed to be a comparator, using the highest feasible dose of *P. knowlesi* sporozoites based on the PkSPZ vialling amount. At exactly 5 days post-sporozoite inoculation, blood was collected for 18S rRNA RT-PCR analysis to assess if parasites had egressed from the liver. Immediately thereafter, animals were euthanized, and livers collected, divided into four lobes, then processed and analyzed to determine parasite burden in each lobe using a slightly modified version of a well-established 18S rRNA RT-PCR biomarker assay [20, 23].

**Fig. 2:**
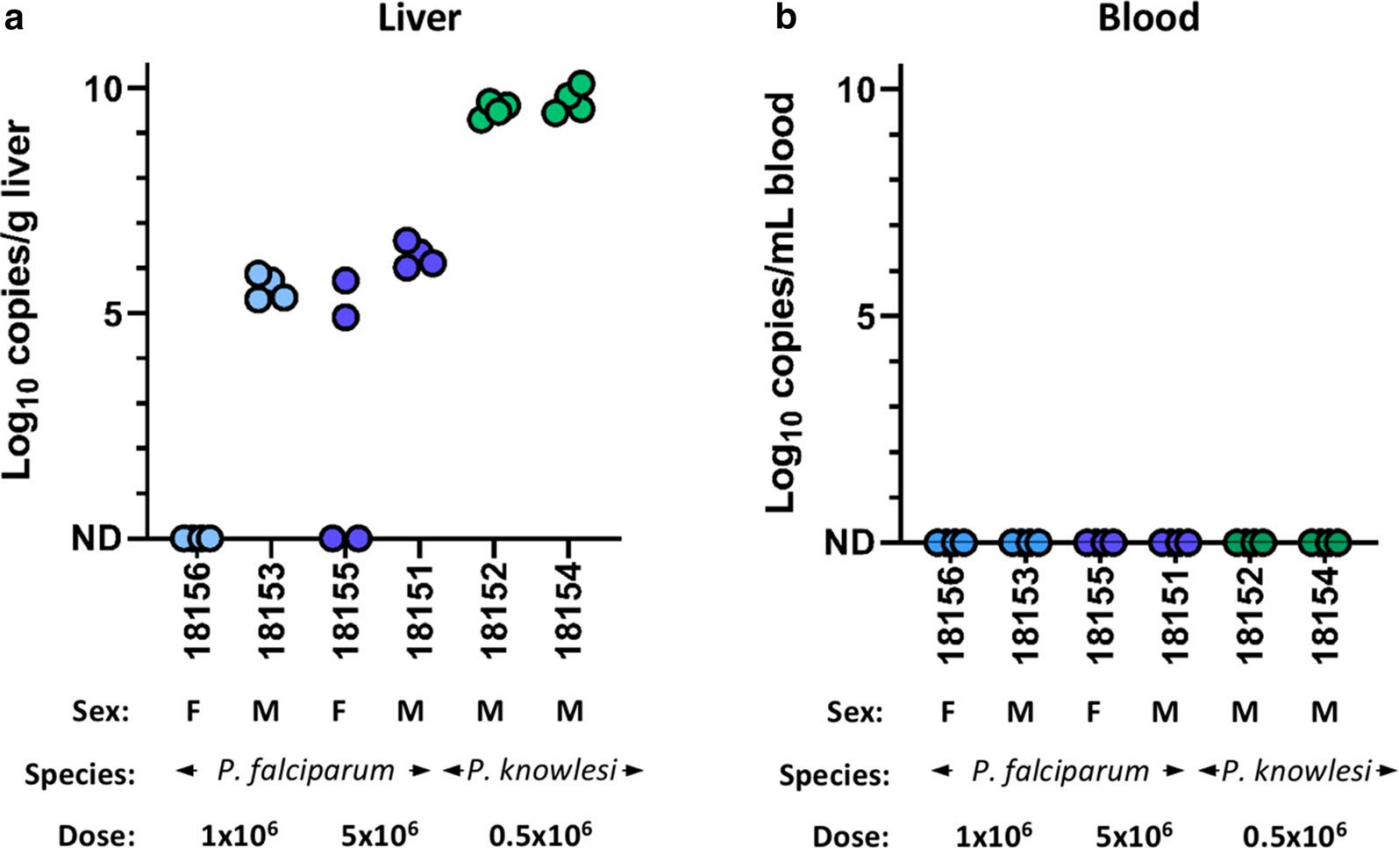
*Plasmodium* 18S rRNA RT-PCR quantification of parasite burden in the liver (**a**) and peripheral blood (**b**) exactly 5 days after intravenous inoculation of PfSPZ or PkSPZ. *P. falciparum* data reported in log_10_ copies of 18S rRNA/g tissue as detected by the *P. falciparum* target. *P. knowlesi* data reported in log_10_ copies of 18S rRNA/g tissue as detected by the pan-*Plasmodium* target. Each data point represents one liver lobe or replicate blood sample. ND, not detected. F: female; M: male. See Table 1 for data by individual lobe

The results for parasite burden in the liver are expressed as log_10_ copies 18S rRNA per g of tissue (Fig. 2a and Table 1). Three of the four animals inoculated with PfSPZ had measurable parasite 18S rRNA in the liver at 5 days post-infection, indicating *P. falciparum* liver burdens following sporozoite administration were somewhat unreliable. The animal with undetectable parasite 18S rRNA had received a dose of 1 × 10^6^ PfSPZ. The other animal that received this dose had detectable parasite 18S rRNA in every liver lobe, with a mean of 5.57 log_10_ copies per g of liver. Both animals that received the higher dose of 5 × 10^6^ PfSPZ had detectable parasite 18S rRNA in the liver, suggesting some impact of sporozoite dose on assay outcome. One animal in this group had 18S rRNA signal in two of four liver lobes, with a mean of 5.32 log_10_ copies per g of liver in the infected lobes, while the other animal had parasite 18S rRNA in every lobe with a mean of 6.27 log_10_ copies per g of liver. Both animals that received PkSPZ had much higher liver parasite 18S rRNA concentrations that were consistent across all four lobes, with means of 9.52 and 9.72 log_10_ copies per g of liver. Parasite 18S rRNA copy number was thus 3–5 log higher in animals that received PkSPZ versus PfSPZ, despite the PkSPZ dose being 2–10 fold lower than in the PfSPZ groups.

**Table 1:** 18S rRNA quantification of liver parasite burden by lobe following intravenous inoculation of PfSPZ or PkSPZ

Animal	Group	LLL	LML	RLL	RML + C	Avg^*^	Age	Sex	Weight (kg)	CRP (mg/L)
A18156	*P. falciparum* (1 × 10^6^)	N.D	N.D	N.D	N.D	N.D	1 year 6 months	Female	2.7	0.5
A18153	*P. falciparum* (1 × 10^6^)	5.31	5.73	5.35	5.88	5.57	1 year 6 months	Male	2.35	< 0.2
A18155	*P. falciparum* (5 × 10^6^)	N.D	N.D	5.73	4.91	5.32	1 year 6 months	Female	2.4	0.3
A18151	*P. falciparum* (5 × 10^6^)	6.02	6.35	6.11	6.61	6.27	1 year 6 months	Male	2.9	0.2
A18152	*P. knowlesi* (0.5 × 10^6^)	9.69	9.30	9.61	9.47	9.52	1 year 6 months	Male	3.15	0.2
A18154	*P. knowlesi* (0.5 × 10^6^)	10.09	9.53	9.82	9.45	9.72	1 year 7 months	Male	1.95	0.7

*P. falciparum* data reported in log_10_ copies of 18S rRNA/g tissue as detected by the *P. falciparum* target. *P. knowlesi* data reported in log_10_ copies of 18S rRNA/g tissue as detected by the Pan *Plasmodium* target

*LML* left medial lobe, *RLL* right lateral lobe, *LLL* left lateral lobe, *RML + C* right medial lobe and caudate lobe, *CRP* C-reactive protein, *N.D* not detected, *F* female, *M* male, Avg* mean of samples where *Plasmodium* 18S rRNA was detected

The magnitude of parasite 18S rRNA in the liver did not appear to correlate with baseline CRP levels or animal body weight (Table 1). While the small sample size of this study precluded any formal correlation analysis, qualitative assessment of the data suggested that these factors were not likely confounding variables. Specifically, for CRP levels, although animals had a range of baseline CRP levels, there was no apparent correlation between this and assay outcome. For body weight, although the smallest animal had the highest 18S rRNA signal following PkSPZ inoculation, the largest animal had the highest 18S rRNA signal following PfSPZ inoculation, giving no clear relationship between body weight and signal. Similarly, for CBC measures, all animals had blood cell counts that were within normal limits based on their age and experience of veterinary staff at the primate center. By contrast, preliminary observations suggest there may be a potential correlation between sex and liver parasite burden following PfSPZ infection. This was evident as both males receiving PfSPZ had detectable 18S rRNA in every liver lobe, but females in the equivalent treatment groups either had no detectable 18S rRNA or signal in only some liver lobes.

The results for parasite burden in the blood are expressed as log_10_ copies 18S rRNA per mL of whole blood (Fig. 2b). Regardless of the treatment group, no animal was found to have detectable parasite 18S rRNA in the blood at 5 days post sporozoite inoculation. This was consistent with the known duration of the *P. falciparum* liver stage in humans and the *P. knowlesi* liver stage in rhesus [21, 22], and the previous data showing no *P. falciparum* 18S rRNA was detectable in the blood of rhesus 6 days post-sporozoite inoculation [20]. This demonstrated that despite the high sporozoite doses given, no detectable parasites had egressed from the liver into the blood at the time of euthanasia and liver collection. Thus, the entirety of the parasite 18S rRNA biomarker signal in the liver as measured above was determined to have originated from parasites in the liver tissue.

## Discussion

Previous studies with the *P. falciparum*/rhesus and *P. knowlesi*/rhesus platforms have demonstrated the value of these models for assessing live-attenuated sporozoite vaccines and elucidating mechanisms of vaccine-induced immunity relevant to humans [15, 17–19]. Since these live-attenuated vaccines must rely on sporozoite invasion for antigen delivery to the liver, studies across these two platforms have only been possible because rhesus have demonstrated some susceptibility to *P. falciparum* sporozoites [15, 16]. However, despite evidence that rhesus are less susceptible to *P. falciparum* versus *P. knowlesi* sporozoite infection, their relative ability to infect and replicate in the rhesus liver has never been directly compared in vivo. Here, using an ultra-sensitive *Plasmodium* 18S rRNA biomarker assay, parasite rRNA copy number in the liver was found to be 3–5 orders of magnitude lower in animals administered PfSPZ versus PkSPZ at 5 days post-infection. While the sample size of this study was small, it was nonetheless sufficient to identify striking trends that have significant relevance for the ongoing use of rhesus in malaria vaccine studies. These findings highlight the fact that there are likely significant differences in host-parasite interactions between *P. falciparum* and *P. knowlesi* in the rhesus liver, which in turn are expected to impact antigen delivery and the immunogenicity of live-attenuated sporozoite vaccines.

The most notable finding of this study is that administration of wild-type cryopreserved *P. falciparum* sporozoites (PfSPZ) resulted in inconsistent, heterogeneous liver stage burdens in vivo. Given that the PfSPZ inoculum was 300 to 1500 times greater than required for 100% blood stage infection in humans [6] and 1000 times the inoculum required to achieve 100% blood stage infection with PkSPZ in rhesus (S. Chakravarty, unpublished), this suggests that *P. falciparum* sporozoites must face considerable barriers to infection in rhesus in vivo. These barriers could include sporozoite exit from the circulation, invasion of hepatocytes, proliferation within hepatocytes, or avoiding immune clearance mechanisms in the liver [24]. The impact of each of these events cannot be distinguished with the current study design, but it is possible to make several further observations. First, although *P. yoelii* sporozoite invasion and development in vivo can be affected by cryopreservation [25], the robust infections seen in the PkSPZ comparator group suggests that cryopreservation was not responsible for the striking difference seen in liver parasite burdens between animals infected with PfSPZ versus PkSPZ in this study. Indeed, although it remains formally possible that *P. falciparum* and *P. knowlesi* sporozoites may tolerate cryopreservation to different extents, the comparable infectivity of cryopreserved PfSPZ in humans and cryopreserved PkSPZ in rhesus described above argue against this as a significant confounding variable. Similarly, as *P. falciparum* sporozoites can invade rhesus hepatocytes in vitro [15, 16], this implies that sporozoite invasion is unlikely to be completely blocked in vivo. On the other hand, as *P. falciparum* liver stage schizont development is only supported at low frequencies in rhesus hepatocytes in vitro [16], this argues that liver stage development may be severely impaired in vivo. Given that avoidance of immune clearance mechanisms by liver stage parasites involves complex and species-specific manipulations of the host cell [26], it is also reasonable to speculate that any *P. falciparum* liver stages that develop in the rhesus liver will have poorer survival than simian-adapted parasite species.

The identification of a potential correlation between sex and liver parasite burden following infection with PfSPZ is of considerable interest. Although there were only two females in this study, the finding that one had no detectable parasite 18S rRNA, and the other had lower signal in fewer liver lobes than the male in the same group was noteworthy. Due to the small sample size of this study, it was not possible to exclude the alternate hypothesis that these findings were due to chance alone, or that despite rigorous procedures, the PfSPZ administration failed in the one animal that did not develop detectable liver stage parasite burden. However, as every effort was made to use age- and weight-matched animals, and to confirm correct administration of PfSPZ into the vein, additional studies in the future may be warranted to determine if this apparent sex-specific decrease in *P. falciparum* liver stage burden is reproducible. While comparatively little is known about sex-specific responses to primary malaria infection, there is an emerging body of literature about sex-specific responses to vaccination [27, 28]. As the animals used here were juveniles, it is unlikely that any sex-specific effects are due to the influence of adult sex hormones on the immune system as documented for live-attenuated sporozoite vaccines in mice [29]. However, other sex-specific immunological differences have been described in human infants and children [28], including in response to the RTS,S malaria vaccine, indicating that the sexes begin to diverge in relevant ways prior to puberty. Given that children are one of the primary target populations for PfSPZ vaccines in endemic areas, it may be beneficial to determine if juvenile rhesus recapitulate these sex-specific differences and responses to malaria vaccination. Further studies to investigate and compare the susceptibility of juvenile versus adult rhesus may also be important to pursue. These studies would enhance the value of rhesus for malaria vaccine testing, and are expected to be key topics for future investigation.

Regardless of the underlying reasons, the heterogeneity of *P. falciparum* burden in the rhesus liver suggests quantification of parasite 18S rRNA is unlikely to provide a useful efficacy measure for vaccines targeting sporozoites and liver stages in the *P. falciparum*/rhesus model. The potential of using the 18S rRNA biomarker assay for this purpose was suggested by a previous study, where rhesus macaques were intravenously administered 6.5 × 10^6^ freshly-purified wild-type *P. falciparum* sporozoites, and parasite 18S rRNA copy number was found to increase between animals sacrificed at days 3 and 6 [20]. This finding suggested that *P. falciparum* 18S rRNA signal could be measured in the rhesus liver over a dynamic range, and hence, that differences in copy number between naïve and vaccinated animals might be used to quantify vaccine efficacy as routinely done in rodent malaria models [30, 31]. Since large doses of freshly-dissected *P. falciparum* sporozoites can be difficult to obtain, lower doses of cryopreserved wild-type sporozoites were assessed here as these would be more ideally suited for vaccine studies. While *P. falciparum* 18S rRNA copy numbers were indeed lower in the current study consistent with the lower sporozoite dose, they were broadly concordant with the previous data, with the day 5 copy numbers measured here similar to or greater than the day 3 copy numbers in the previous study [20]. However, neither dose of PfSPZ resulted in consistent burdens, meaning that parasite 18S rRNA copy number measurements would be highly subject to where in the liver was sampled, or necessitate sampling of the entire liver as performed here. Unfortunately, this may be impractical for vaccine studies since protocols for simultaneous processing of liver samples for 18S rRNA and immunological assays are yet to be developed for rhesus, and costs and ethical concerns would preclude most researchers from performing immunogenicity and efficacy studies in parallel cohorts of rhesus. Taken together, this ultimately suggests that quantification of parasite 18S rRNA in the liver is unlikely to be a feasible way to assess vaccine efficacy in the *P. falciparum*/rhesus model.

Finally, this study has several important implications for research involving live-attenuated parasite vaccines in rhesus. Live-attenuated vaccines that rely on sporozoite invasion of the liver encompass a spectrum of vaccine concepts that include radiation-attenuated sporozoites, late-arresting genetically-attenuated parasites, and chemically-attenuated parasites [1], the latter including the PfSPZ-CVac chemoprophylaxis vaccine [6]. These vaccines differ in their methods of attenuation, the extent of parasite development in the liver, and the corresponding breadth of antigens delivered to the immune system [1]. Given the ability to meaningfully evaluate these vaccines is critically dependent on to what extent rhesus can support sporozoite invasion and liver stage development, the quantitative data presented here offer new insight into how the *P. falciparum/*rhesus and *P. knowlesi/*rhesus platforms can best be used to evaluate these vaccines. For radiation-attenuated sporozoites that arrest early in the liver, previous studies have already demonstrated that both the *P. falciparum* and *P. knowlesi* platforms can provide highly valuable immunogenicity data about these vaccines [15, 17, 18]. Given the relatively poor susceptibility of rhesus to PfSPZ revealed here, it may be necessary to re-interpret some of the data from past studies of *P. falciparum* attenuated sporozoite vaccines in rhesus to take this decreased susceptibility into consideration. However, this certainly does not impact the validity of using rhesus to assess antibody and T cell responses induced by *P. falciparum* attenuated sporozoite vaccines, but rather serves to refine understanding of the model and potentially improve how findings from these studies are extrapolated to humans. Equally, as there is evidence that *P. falciparum* sporozoites can invade rhesus hepatocytes in vitro [15, 16] and in vivo [20], this also suggests that both platforms can be used to study responses to pre-formed antigens induced by radiation-attenuated sporozoite vaccines [32], with the caveat that the decreased susceptibility of rhesus to *P. falciparum* sporozoites should again be taken into consideration. The decreased susceptibility of the rhesus liver to *P. falciparum* versus *P. knowlesi* sporozoites should also be considered for making comparisons across platforms, as it is expected to impact antigen load, antigen distribution across the liver, and the expression of liver stage-specific antigens. Conversely, for genetically- or chemically-attenuated parasites vaccines that arrest at later stages of development, the data presented here indicate that *P. falciparum*/rhesus model is unsuitable for evaluation of these vaccines, and suggests such vaccines should instead be assessed using alternative platforms.

## Conclusions

This study provides the first direct quantitative comparison of the relative susceptibility of the rhesus liver to wild-type *P. falciparum* versus *P. knowlesi* sporozoites in vivo. This data will help guide pre-clinical studies of live-attenuated parasite vaccines in the *P. falciparum*/rhesus and *P. knowlesi*/rhesus models and enable better integration data from vaccine studies across platforms.

## Data Availability

The datasets used and/or analyzed during the current study are available from the corresponding author on reasonable request.

## Abbreviations

CBC: Complete blood count
CRP: C-reactive protein
DVI: Direct venous inoculation
LLL: Left lateral lobe
LML: Left medial lobe
PCR: Polymerase chain reaction
PfSPZ: *P. falciparum* sporozoites
PkSPZ: *P. knowlesi* sporozoites
qRT-PCR: Quantitative reverse transcription PCR
RLL: Right lateral lobe
RML + C: Right medial lobe plus caudate lobe
rRNA: Ribosomal RNA
WaNPRC: Washington National Primate Research Center
